# Abdominal Imaging in ADPKD: Beyond Total Kidney Volume

**DOI:** 10.3390/jcm12155133

**Published:** 2023-08-05

**Authors:** Anna Caroli, Timothy L. Kline

**Affiliations:** 1 Bioengineering Department, Istituto di Ricerche Farmacologiche Mario Negri IRCCS, 24020 Ranica, BG, Italy; 2 Department of Radiology, Mayo Clinic, Rochester, MN 55905, USA; kline.timothy@mayo.edu

**Keywords:** autosomal dominant polycystic kidney disease, total kidney volume, cyst volume, non-cystic tissue, magnetic resonance imaging, segmentation, artificial intelligence

## Abstract

In the context of autosomal dominant polycystic kidney disease (ADPKD), measurement of the total kidney volume (TKV) is crucial. It acts as a marker for tracking disease progression, and evaluating the effectiveness of treatment strategies. The TKV has also been recognized as an enrichment biomarker and a possible surrogate endpoint in clinical trials. Several imaging modalities and methods are available to calculate the TKV, and the choice depends on the purpose of use. Technological advancements have made it possible to accurately assess the cyst burden, which can be crucial to assessing the disease state and helping to identify rapid progressors. Moreover, the development of automated algorithms has increased the efficiency of total kidney and cyst volume measurements. Beyond these measurements, the quantification and characterization of non-cystic kidney tissue shows potential for stratifying ADPKD patients early on, monitoring disease progression, and possibly predicting renal function loss. A broad spectrum of radiological imaging techniques are available to characterize the kidney tissue, showing promise when it comes to non-invasively picking up the early signs of ADPKD progression. Radiomics have been used to extract textural features from ADPKD images, providing valuable information about the heterogeneity of the cystic and non-cystic components. This review provides an overview of ADPKD imaging biomarkers, focusing on the quantification methods, potential, and necessary steps toward a successful translation to clinical practice.

## 1. Introduction

Autosomal dominant polycystic kidney disease (ADPKD) is the most common hereditary renal disease, and the fourth-leading cause of end-stage renal disease (ESRD) in adults [1,2]. In ADPKD, the renal function remains normal or near normal for decades, before starting to decline inexorably, with approximately 50% of ADPKD patients needing renal replacement therapy by the age of 60 years [3,4].

The clinical hallmark of the ADPKD phenotype is the progressive enlargement of the kidneys [5], caused by the formation and progressive expansion of multiple fluid-filled cysts originating from the tubule wall, leading to the compression and obstruction of adjacent nephrons, and to injury in the normal parenchyma, despite an apparently normal renal function [6].

We urgently need early renal disease biomarkers that can not only detect current damage, but also potentially predict the course of the disease. This need persists even when the renal function, gauged using serum creatinine levels or the estimated glomerular filtration rate (eGFR), appears normal, or is relatively maintained. These biomarkers are crucial for clinical monitoring, and for evaluating new treatment approaches during clinical trials.

**Figure 1:**
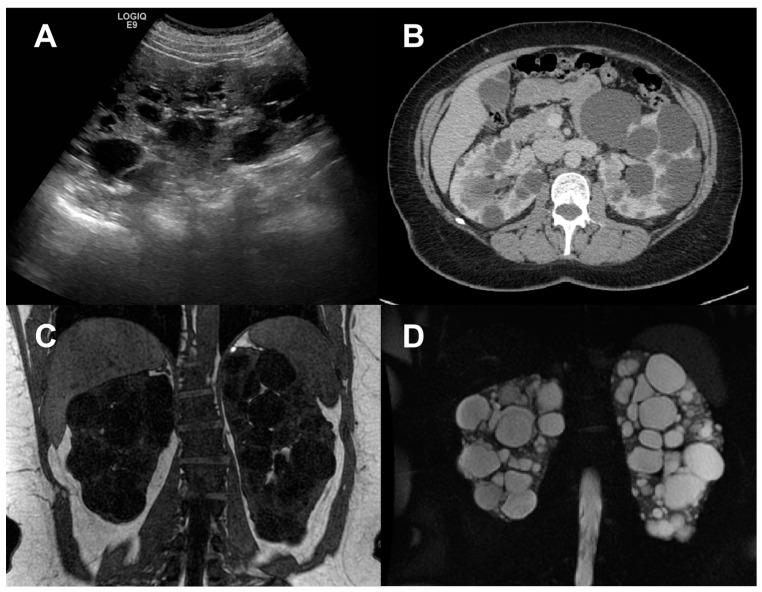
Anatomical imaging of the polycystic kidney. Representative B-mode US image (**A**), contrast-enhanced CT image (**B**), and anatomical MRI, obtained via T1-weighted (**C**) and T2-weighted (**D**) sequences in human patients with autosomal dominant polycystic kidney disease. All techniques illustrate the presence of enlarged echogenic kidneys, and fluid-filled cysts in the kidney parenchyma.

As renal enlargement is the underlying macroscopic process, ultrasonography (US), computerized tomography (CT), and magnetic resonance imaging (MRI) have been used for many years to quantify kidney disease progression in ADPKD (Figure 1) [3,7,8,9,10], recently allowing us to also investigate the relationship between the kidney or cyst volume increase, and renal function decline in the polycystic kidney [11,12,13].

Abdominal imaging also allows the investigation of extra-renal manifestations of ADPKD, including liver cysts [14], pancreatic cysts [15], seminal megavesicles [16], and splenomegaly [17], as well as pleural [18] and pericardial effusions [19].

In this review, we provide an overview of the diagnostic imaging state of the art in ADPKD, focusing on the relevance of different renal imaging biomarkers, from the most established—the total kidney and cyst volume—up to the most innovative ones, which will likely allow the monitoring of the onset and progression of ADPKD from the earliest stages of the disease, and the monitoring of the response to targeted therapy, even before the renal function declines. The imaging biomarker quantification methods will be reviewed, including the latest artificial intelligence (AI) developments, and the potential of multiparametric approaches will be discussed.

## 2. Total Kidney Volume

An increase in the total kidney volume is acknowledged by the scientific community as the dominant feature in ADPKD progression [11]. Back in 2000, two independent longitudinal pilot studies provided preliminary evidence of a TKV increase from year to year in ADPKD patients, suggesting that the rate of TKV growth, reflecting the enlargement of cysts, might be a sensitive metric of disease progression [20,21]. From then on, these results have been confirmed by several other studies [11].

In 2006, the Consortium for Radiologic Imaging Studies of Polycystic Kidney Disease (CRISP) performed a longitudinal study in 241 patients with ADPKD and a baseline eGFR >70 mL/min, exploring the hypothesis that the TKV is a sensitive indicator of disease progression [3,5]. Tracking the progress of patients, including frequent GFR measurements using iothalamate clearance and regular kidney MRIs, revealed several key points: (1) the TKV demonstrated an exponential growth over time, (2) the rate of kidney growth varied among patients, (3) a faster decline in the GFR was found to be linked with a higher kidney growth rate, and (4) patients with PKD2 mutations showed a slower increase in kidney volume, and a slower decline in the GFR, compared to those with PKD1 mutations, likely due to fewer cyst formations [4,12,13,22]. Taken all together, these findings support the role of the sequential measurement of the TKV as a quantifiable index of disease progression. In addition, the baseline TKV was found to predict GFR decline within eight years [4].

In 2015, Irazabal et al. proposed a model to predict GFR decline, using a single height-adjusted TKV (HtTKV) measurement, alongside age [23,24]. According to this model (the Mayo Imaging Classification), based on the assumptions of exponential kidney growth, and a fairly stable kidney growth rate over years [25], typical ADPKD patients can be divided into five classes (1A-E), characterized by their different renal function decline rates. This classification method, easily applied using the Mayo Clinic website (https://www.mayo.edu/research/documents/pkd-center-adpkd-classification/doc-20094754, accessed on 25 May 2023), was validated in HALT-PKD [24,26] and TEMPO [27] studies, as well as in independent cohorts [28,29], showing its utility as an early marker of disease evolution in ADPKD patients.

The Mayo Imaging Classification was developed to predict progression more accurately, and allow for better risk stratification among patients affected by ADPKD. Before this classification was introduced, the disease’s heterogeneity meant that it was challenging to predict individual patients’ progression. This classification system acknowledges the significant role that the TKV and age play in disease progression, with a larger TKV and younger age being associated with more rapid disease progression. For instance, a young patient with significantly enlarged kidneys (Class 1B) may be at higher risk of progression to end-stage renal disease than an older patient with smaller kidneys (Class 1A).

Moreover, this classification can guide therapeutic decisions, as patients with rapidly growing kidneys (Classes 1C to 1E) could be considered for early therapeutic intervention. On the other hand, those in Class 1A, with a slower disease progression, could be managed conservatively, with monitoring and symptomatic treatment.

The Mayo Imaging Classification, therefore, represents a significant step toward personalized medicine in ADPKD, allowing clinicians to tailor treatments based on individual risk profiles. It underscores the importance of the TKV measurement in the management of ADPKD. However, further research is needed to refine this system, and improve the predictive accuracy, potentially by incorporating other factors, such as genetic variations or more advanced imaging biomarkers.

The TKV can also serve as an indicator to evaluate the efficacy of candidate therapeutic agents that target cyst formation and growth in ADPKD patients [30,31,32,33,34,35,36,37,38]. Clinical studies have highlighted that drug treatment in these patients could limit kidney enlargement earlier, and to a greater extent, than slowing the renal function decline [11].

Finally, the TKV has been recently recognized by the EU and US regulatory agencies as an enrichment biomarker, and a possible surrogate endpoint. In response to a request from the FDA, the Polycystic Kidney Disease Outcomes Consortium (PKDOC) gathered TKV data from more than two thousand patients with ADPKD, to track the decline in the eGFR as a function of the baseline TKV [39]. As a result of the PKDOC findings, in 2015, the US Food and Drug Administration (FDA) published a letter of support, and the European Medicines Agency (EMA) published a positive qualification opinion for the TKV as an exploratory prognostic biomarker for enrichment in ADPKD clinical trials [40,41]. In 2016, the FDA qualified the TKV as a prognostic biomarker and, in 2018, it further designated the TKV as a reasonably likely surrogate endpoint [42].

**Figure 2:**
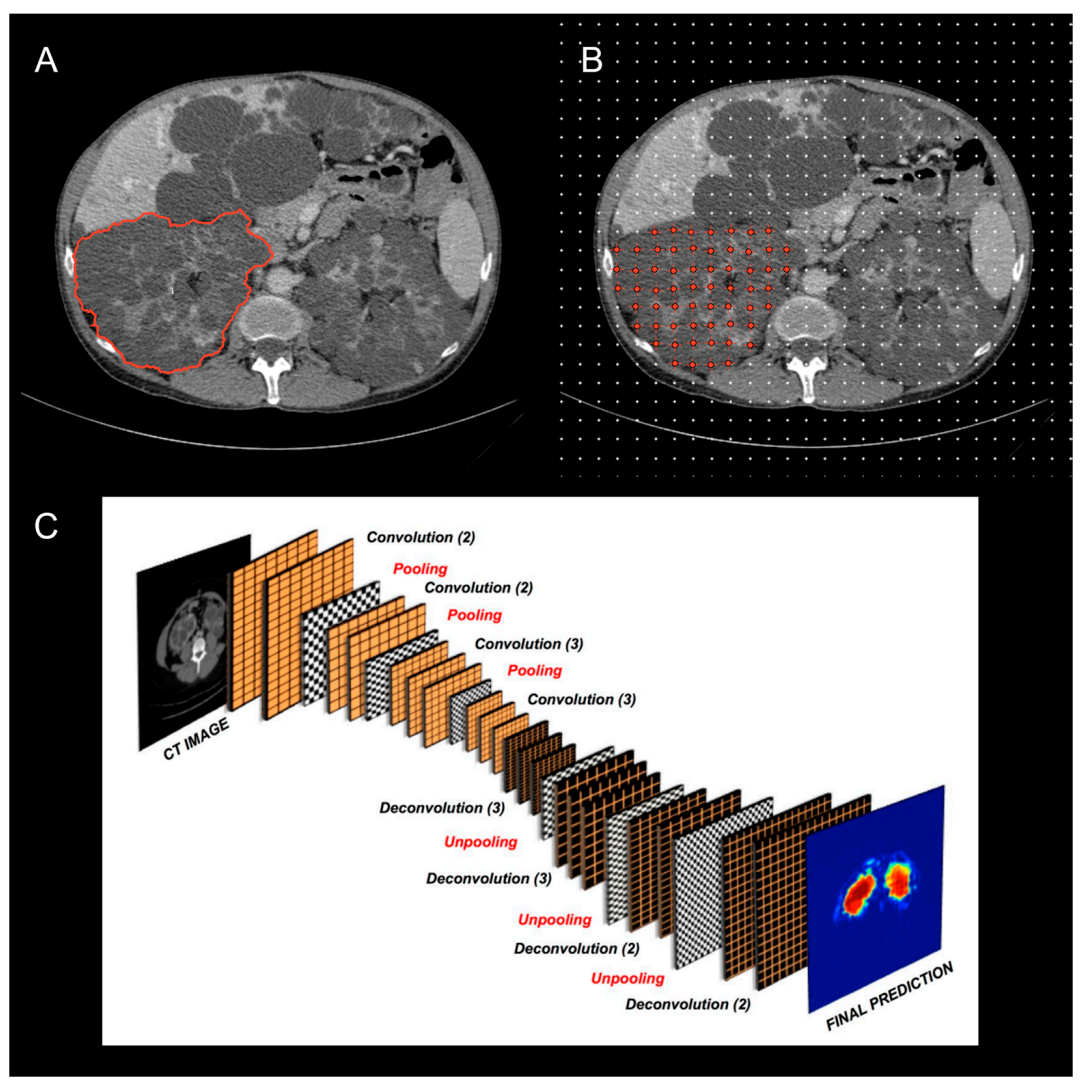
Example of methods available to outline the kidneys and quantify the total kidney volume on CT scans: (**A**) manual planimetry; (**B**) stereology; (**C**) automatic segmentation using fully convolutional neural networks. The figure was modified from Sharma K. et al. Kidney volume measurement methods for clinical studies on autosomal dominant polycystic kidney disease [43] (**A**,**B**); and Sharma K. et al. Automatic Segmentation of Kidneys using Deep Learning for Total Kidney Volume Quantification in Autosomal Dominant Polycystic Kidney Disease [48] (**C**), under the terms of the CC-BY 4.0 license.

The current and potential use of the TKV has drawn attention to the variety of imaging modalities and methods employed for calculating the TKV across ADPKD studies (Figure 2) [43,44,45]. The TKV can be reliably measured via either MRI or CT, with methods that differ in complexity, the time required, accuracy, and precision. As of now, the most commonly used methods include whole kidney manual contouring (hereafter named as planimetry) and stereology (grid point counting over the kidney) [46]. As these methods are time consuming, simpler and faster methods using a mid-slice approach [47] or an ellipsoid equation [23] have been proposed, to shorten the time required to estimate the TKV.

The choice of one method rather than another depends on the purpose of use. Simplified methods are equivalent to manual planimetry in the ADPKD classification of disease severity, or in identifying patients with a high likelihood of rapid disease progression for enrolment in clinical studies [23,49,50]. Contrarily, when the TKV is used to monitor disease progression, or as a clinical trial endpoint, the TKV measurements must be more precise and accurate, to effectively detect small changes over time intervals as short as 6 months or 1 year. The use of precise measurements is also necessary to limit the number of patients to enroll, thus making clinical studies more feasible and significant. The mid-slice and ellipsoid methods demonstrated a poor reproducibility and low precision and accuracy, compared with manual planimetry or stereology [43,51]. In addition, simplified methods, due to the high variability in estimating the TKV, would require a 4-fold larger sample size than manual planimetry to find a significant difference between the TKV changes occurring in two treatment groups [43].

Despite being precise and accurate, manual planimetry requires 20 to 40 min on average. To overcome the time requirement and operator-dependency limitations, completely automated methods are desirable.

Automatic quantification techniques for the TKV involve the utilization of computer algorithms to accurately, efficiently, and reproducibly measure the TKV from different types of imaging modalities, such as CT, MRI, or US. These techniques offer several advantages compared to manual methods, including an enhanced precision, reduced variability, and improved expediency. By leveraging advanced algorithms and image processing capabilities, automatic quantification techniques enable more precise and consistent measurements of the TKV, minimizing human error and subjective interpretation. Additionally, these automated methods allow for faster analysis, saving valuable time in research and clinical settings. The application of automatic quantification techniques contributes to the standardization of TKV measurements, facilitating the reliable monitoring and assessment of kidney volume changes.

Prior to 2017, automated methods required some level of human intervention (i.e., semi-supervised [52,53]) or reliance on comparative studies (i.e., atlas-based and or registration-based approaches [54]). In 2017, Kline et al. used MRI to develop a fully automated deep-learning-based approach to performing kidney segmentations [55]. The authors highlighted that current image processing tasks, such as organ segmentation and classification, require large datasets for effective deep-learning training. They created a unique database of MRI scans and reference standard segmentations of polycystic kidneys to aid this process. They used 2000 cases for the training and validation of the method, with an additional 400 cases for testing. Their multi-observer ensemble method achieved a mean percent volume difference of 0.68 ± 2.2%, compared to the reference standard segmentations. The authors suggest that this fully automated method performs at a level comparable with human interobserver variability, and could replace manual kidney segmentation.

In the same year, Sharma et al. proposed an automated segmentation method based on deep learning [48]. This method was developed for TKV computation on CT datasets among ADPKD patients who exhibited mild to moderate or severe renal insufficiency. The technique was trained on 165 cases, and tested on 79 cases, covering a wide range of TKVs (321.2–14,670.7 mL). The method achieved an overall mean Dice similarity coefficient of 0.86 ± 0.07, indicating a high level of agreement between the automated segmentations, and manual segmentations performed by clinical experts. Moreover, the segmented kidney volume measurements showed a mean correlation coefficient of 0.98 (*p* < 0.001) in the entire test set. In conclusion, the proposed method offered fast and reproducible kidney volume measurements, aligning closely with manual segmentations conducted by clinical experts.

Since this report, many studies have also demonstrated the power of AI-based approaches to solving the task of automated segmentation in images of patients affected by ADPKD. By leveraging advanced imaging technologies and computational algorithms, researchers have successfully developed methods that not only streamline the measurement process, but also offer an improved accuracy compared to traditional manual approaches. These advancements are vital in enhancing the efficiency and reliability of TKV assessments, which in turn can contribute to better monitoring, diagnosis, and treatment evaluation in various kidney-related conditions and diseases. Recent work has also used 3D US to perform the automated segmentation of kidneys, and calculate the TKV [56].

In addition to improving the accuracy and efficiency of the TKV measurement, automatic quantification techniques also have the potential to reduce the interobserver variability. This is particularly important in ADPKD, where the TKV is used as a prognostic marker, and must be measured consistently over time. Using an automated analysis approach, Edwards et al. evaluated the tracking of kidney growth in clinical trials for ADPKD, and found that the transition between different readers in a previously published clinical trial led to a bias/shift in the TKV measurements and growth rates, highlighting the importance of standardized methods to minimize errors in serial TKV measurements [44].

Overall, automatic quantification techniques for the TKV offer a promising solution for the efficient and accurate measurement of the TKV in patients affected by ADPKD. These techniques have most recently been incorporated into clinical workflows [57,58], making clinical adoption highly likely in the near future [59].

There are several considerations for the frequency of TKV measurement. Firstly, a too-frequent assessment may expose patients to unnecessary testing procedures, such as MRI or CT scans, which have associated risks and costs. Secondly, assessments that are too infrequent may miss crucial disease progression, which could have been targeted for early intervention. The optimal frequency of TKV evaluation will depend on several factors:

- Disease severity and progression: A more aggressive and fast-progressing disease may warrant more frequent monitoring.
- Age and sex of the patient: The disease progression can vary significantly between different age groups, and between men and women.
- Treatment regimen: Patients on specific therapies may need more frequent TKV monitoring, to evaluate treatment efficacy.
- Resource availability: The availability of resources, such as diagnostic imaging services, trained personnel, and financial resources can affect the frequency of TKV measurement.

Future studies should consider these factors when determining the TKV measurement frequency. Randomized controlled trials that compare different monitoring frequencies may help establish a standardized protocol. Additionally, non-invasive and less costly ways to measure the TKV could be a promising research direction, making it easier to conduct frequent assessments.

One such approach that is beginning to be investigated is the use of three-dimensional ultrasound (3D US). This technique is non-invasive, radiation-free, less expensive, and widely available, making it a potentially more accessible and repeatable option for TKV assessment. However, the implementation of 3D US as a primary tool for TKV measurement in ADPKD patients is not without its challenges:

- Technical expertise and standardization: It requires a high level of skill to capture and interpret 3D US images correctly. These skills are not as universally prevalent among clinicians as the expertise for 2D US, which can affect the availability and consistency of 3D US-based TKV measurements. The standardization of the technique across different operators and machines is also necessary, to ensure accurate and comparable measurements.
- Accuracy and reliability: While studies have shown promising results, further validation is needed to establish 3D US’s accuracy and reliability compared to MRI and CT, particularly in patients with very large kidneys or a complex cyst morphology.
- Processing time: While obtaining 3D US images may be quicker than with MRI or CT, processing and interpreting these images can be time-consuming. Advances in automated or semi-automated interpretation software could help address this challenge.

Therefore, while the potential for 3D US in TKV measurement is significant, further research is necessary to fully explore and address these challenges. Ideally, large-scale studies comparing 3D US with MRI or CT for performing TKV measurements in ADPKD patients would help to establish its effectiveness, and inform practice guidelines.

## 3. Cyst Volume

**Figure 3:**
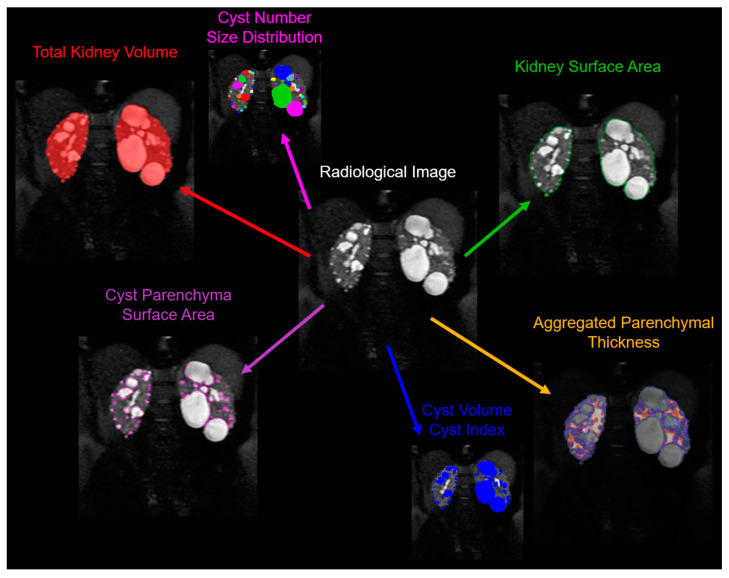
Example of a multifaceted analysis of ADPKD pathology derived from a single radiological image. This representative image depicts the comprehensive measurement parameters that can be employed to provide in-depth information about kidney enlargement and the cyst burden in ADPKD. Clearly delineated are various parameters, including: the total kidney volume—indicating the comprehensive volume of the kidney, inclusive of cysts and the parenchyma; the cyst number and size distribution—signifying the count and varying dimensions of individual cysts present in the kidney; the kidney surface area—the measure of the external boundary of the kidney; the aggregated parenchymal thickness—denoting the cumulative thickness of the renal parenchyma; the cyst volume—indicating the space occupied by each individual cyst; the cyst index—a ratio demonstrating the proportion of the kidney volume taken up by cysts; and the cyst parenchyma surface area—representing the interface between the cystic and non-cystic renal tissue. This multiparametric assessment provides a granular view of the ADPKD-affected kidney, which aids in understanding disease progression, and may guide clinical decision making and therapeutic decisions.

Although the TKV, which includes both cystic and non-cystic components, has been identified as a reliable biomarker of disease progression, the assessment of the cyst burden by itself is likely a more detailed characterization of the state of disease (Figure 3). Several studies have shown a strong correlation between cyst volumes and ADPKD progression.

In 2006, Harris et al. used serial renal magnetic resonance imaging data from the Consortium of Radiologic Imaging Study of PKD (CRISP), and found that cystic expansion occurs at a consistent rate per individual, but is heterogeneous across the population [60]. Additionally, larger kidneys were associated with a more rapid disease progression. In this study, the gene type (PKD1 or PKD2) was determined in 183 families, encompassing 219 cases. The majority, 156 (85.2%), had PKD1, while 27 (14.8%) had PKD2. The researchers found that PKD1 kidneys were significantly larger, but the rate of cystic growth was not different between PKD1 (5.68% per year) and PKD2 (4.82% per year) (*p* = 0.24). The number of cysts increased with age, and more cysts were detected in PKD1 kidneys (*p* < 0.0001). The study concludes that PKD1 is more severe, not because the cysts grow faster, but because more cysts develop earlier. This suggests that the disease gene is implicated in cyst initiation, but not in cyst expansion. Technological advancements have significantly improved the precision of cyst volume measurements. High-resolution imaging modalities, such as CT and MRI, are commonly used for this purpose. The development of automated and semi-automated algorithms for image analysis has further increased the accuracy and efficiency of volume measurements, enabling the longitudinal analysis of cyst volume changes over time, which is crucial for monitoring the progression of ADPKD.

In 2013, Bae et al. assessed the performance of a semi-automated method for segmenting individual renal cysts from MRI scans in patients with ADPKD [61]. Utilizing a morphologic watershed technique with a shape-detection level set, the method was applied to T2-weighted MRI scans from 20 patients (40 kidneys) with a mild to moderate renal cyst burden. The performance of the semi-automated method was evaluated against manual counting, and a region-based thresholding method. The results showed a high intraclass correlation (ICC, 0.99 for the cyst count and 1.00 for the cyst volume), with a relative bias of 0.3% for the cyst count and <10% for the cyst volume. The findings indicate that the semi-automated method can accurately segment individual renal cysts, offering a reliable quantitative indicator of disease severity in the early and moderate stages of ADPKD.

The following year, the same group developed and evaluated a semi-automated technique for segmenting and counting individual renal cysts from mid-slice MRI (i.e., the coronal slice with the maximum kidney area) in patients with ADPKD [62]. This method was applied to images from 241 subjects with ADPKD. The researchers compared the semi-automated cyst counts to the manual cyst counts, using ICC and Bland–Altman plots. The results showed successful cyst segmentation in all 241 cases, and a good correlation between the two methods (ICC = 0.96 for either kidney). However, there was a small average difference in the cyst count, with the semi-automated method having a slightly higher count for the right kidney, and the manual method having a slightly higher count for the left kidney. Notably, in 2.5% of participants, the difference in the total cyst count was more than 15. Nonetheless, the study concluded that the semi-automated method can be used as a quantitative tool for characterizing and tracking ADPKD progression.

In 2020, Bae et al. proposed the exclusion of exophytic cysts, portending a favourable prognosis, from HtTKV computation, to facilitate the inclusion of class 2 patients, and the reclassification of class 1 patients in the Mayo imaging classification model, providing further evidence in support of the importance of segmenting individual cysts [63].

In 2021, Kline et al. developed and tested a fully automated method for analyzing kidney cysts in patients with ADPKD, using semantic segmentation [64]. This method was trained and validated on a set of 60 MRI T2-weighted scans, using a three-fold cross-validation approach to train three models. An ensemble model was then tested against manual segmentations made by two readers. The automated approach performed comparably to the variability between the two human readers. It achieved a Dice coefficient (a measure of segmentation similarity) of 0.86 and 0.84 versus the two readers, compared to the interobserver Dice of 0.86. In terms of the total cyst volume (TCV), the automated method showed a percent difference of 3.9% and 8.0% versus the two readers, whereas the interobserver variability was −2.0%.

In 2022, Rombolotti et al. developed a deep-learning model based on the U-Net architecture for kidney and cyst segmentation in preclinical micro-CT images [65]. The aim was to determine which networks performed best on contrast-enhanced micro-CT images from normal rats, and rats with ADPKD. They evaluated the network performance, using the intersection-over-union and Dice coefficients. The study found that the highest-performing networks were the U-Net with a batch normalization layer applied after each pair of 3 × 3 convolutions, and the U-Net where convolutional layers were replaced by inception blocks. These networks accurately provided cyst-to-kidney volume ratios from segmented images, an important metric for monitoring disease progression. The segmentation performance was also stable, regardless of variations in the training image set.

Raj et al., also in 2022, developed advanced machine-learning methods to improve the estimation of the total kidney volume (TKV), a critical biomarker for the early detection of autosomal dominant polycystic kidney disease (ADPKD) [66]. They incorporated three attention mechanisms into the existing U-Net model, implemented a cosine-loss function designed for small datasets, and utilized sharpness aware minimization (SAM) to enhance the network generalizability. Their techniques significantly outperformed the conventional kidney segmentation U-Net, achieving a dice score (DSC) of 0.918, a mean symmetric surface distance (MSSD) of 1.20 mm, a mean TKV difference of −1.72%, and R2 of 0.96, using just 100 MRI datasets for both training and testing. Testing with four ensembles further improved the DSC to 0.922, and the MSSD to 1.09 mm, surpassing the performance of the best individual network.

In 2023, Cui et al. introduced a novel, efficient method, named HUNet, for enhancing the measurement of the TKV [67]. HUNet, a multi-module hybrid U-shape segmentation method, incorporates wavelet pooling, cascade residual, and efficient multi-head self-attention, within the U-shape structure. Wavelet pooling replaces traditional down-sampling to minimize detail loss, cascade residual modules enhance the feature reusability, and efficient multi-head self-attention captures global multi-scale information effectively. During the decoding process, a weighted average is used to determine the total loss value from each decoder’s corresponding loss value. Trained and tested on an ADPKD dataset, the method displayed an impressive accuracy, achieving a Dice similarity coefficient of 0.915 relative to manual segmentation, and a negligible 0.4% difference between the TKV values obtained using the manual and HUNet methods. This research represents a significant advancement in the rapid and precise measurement of the TKV in ADPKD.

Beyond semantic segmentation, a recent study by Gregory et al. [68] developed an instance-based segmentation method to not only characterize the cyst volume, but also the cyst number, cyst size distributions, and cyst spatial relationships and location. Using a convolutional neural network (CNN), they trained the system to learn and distinguish cyst edges and cyst cores. To train this model, they used 30 MRI scans, and validated it on 10 MRI scans, with a fourfold cross-validation procedure. The final ensemble model was then tested on 20 MRI data that had not been used during training/validation. The test set results were compared with the segmentations conducted by two readers. The developed model achieved impressive results, with an average R^2^ value of 0.94 for the cyst count, 1.00 for the total cyst volume, and 0.94 for the cystic index, defined as the percentage of cyst volume over total kidney volume. It also achieved an average Dice coefficient (a measure of similarity) of 0.85, demonstrating the feasibility of automatic cyst segmentations in ADPKD patients. This approach could provide a more detailed insight into the progression of the disease in individual patients.

This model was used by the team in a follow-up study, to look at the utility of the new metrics [69]. The main motivation for this study was that patients with similar TKVs can have significantly different clinical presentations, which necessitates a more nuanced approach. The researchers developed a technique to individually segment and quantify cysts in the kidneys, providing biometric parameters such as the cyst volume, cyst number, parenchyma volume, and cyst parenchyma surface area. These data were collected and analyzed from the CRISP study. The findings revealed that the total cyst number and cyst parenchyma surface area were superior predictors of the rate of the decline in the estimated glomerular filtration rate (a measure of kidney function), kidney failure, and chronic kidney disease stages 3A, 3B, and 4, compared to the TKV. The study also demonstrated that certain presentations, such as having a few large cysts that contribute significantly to the overall kidney volume, could be more accurately stratified for outcome predictions using the new technique. This research suggests that these newly identified image biomarkers, which can be obtained automatically, may greatly aid future studies and the clinical management of patients with ADPKD.

Beyond regular fluid-filled cysts, hemorrhagic and/or complex cysts have recently been shown to play a role in ADPKD progression, being an independent predictor of eGFR decline, alongside other MR biomarkers [70].

In conclusion, cyst volume measurement and characterization are crucial aspects of monitoring the progression of ADPKD. Automated methods for measuring the cyst volume provide a reliable and efficient alternative to manual methods, offering several advantages, including an improved accuracy, faster measurement time, and reduced interobserver variability. Thus, automated methods are an essential tool in ADPKD management, and should be incorporated into routine clinical practice.

## 4. Non-Cystic Tissue

**Figure 4:**
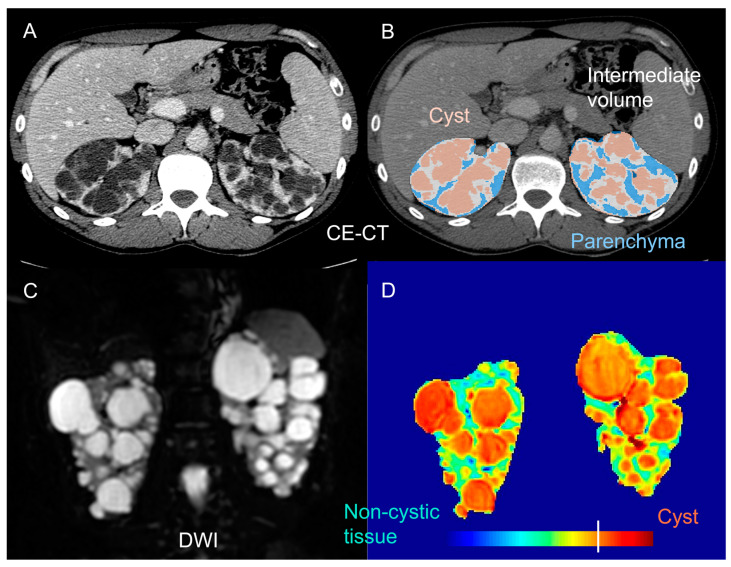
Examples of imaging techniques allowing the quantification and characterization of non-cystic kidney tissue in ADPKD. **Top row**: the automatic segmentation of cysts, intermediate volume (non-cystic fibrotic component), and residual parenchyma (**B**) on a contrast-enhanced (CE) CT scan (**A**), as described in [71]. **Bottom row**: DWI b0 scan (**C**) and pertinent DWI-based diffusivity (**D**) map (**D**), clearly distinguishing cysts and non-cystic tissue, and allowing the characterization of the non-cystic component, as described in [75].

Beyond the total kidney and cyst volume, the quantification and characterization of the non-cystic kidney tissue component (Figure 4) has recently shown the potential to stratify ADPKD patients early, monitor disease progression, and possibly predict renal function loss [71,72,73,74].

In 2006, Antiga et al. used contrast-enhanced (CE) CT imaging to separate and characterize different kidney tissue components in ADPKD patients with normal renal function to severe renal dysfunction [71]. In addition to cysts, appearing uniformly dark, and the renal parenchyma, appearing bright and assumed to represent functioning tissue, a third tissue component, called “intermediate volume”, was classified, to account for the presence of regions appearing hypoenhanced, compared to typical vascularized tissue. The intermediate volume showed a specific image intensity on CE-CT (ranging from 50 to 80 HU), independently of the patient size, and of the amount of residual parenchyma, ensuring the possibility of accurately identifying it. Significant inverse correlations were found between the GFR, and the ratio between the intermediate and parenchymal volume, as well as between changes in the GFR, and the relative intermediate volume. In the limited group of patients studied, there was no correlation between the volumes of cysts or parenchyma, and the GFR or its decline. This implies that an increase in the size of the cysts may not necessarily lead to a deterioration in kidney function. Instead, smaller-scale changes may play a more direct role in the loss of renal function.

In 2011, Caroli et al. investigated the structural nature of the previously identified intermediate volume on kidneys excised from three ADPKD patients with end-stage renal disease, already on hemodialysis, who underwent CE-CT before surgery [72]. Histological samples of tissue corresponding to the intermediate volume on CE-CT images were consistently characterized by sparse dilated tubules, microcysts, and peritubular interstitial fibrosis [72], in line with previous histological findings showing the presence of interstitial fibrosis in ADPKD [76,77,78,79,80,81,82,83,84], in spite of its controversial role [85]. From an imaging standpoint, the hypo-enhancement of the intermediate volume on CE-CT could reflect the uptake of the contrast agent in the sparse, dilated tubules, and the reduced uptake throughout the fibrotic tissue, where peritubular capillaries are stretched via secondary tissue hypoperfusion, globally leading to a loss of enhancement on CE-CT images on the macroscale.

Tubular atrophy and interstitial fibrosis are known to occur concurrently with cyst development, even in ADPKD patients with a normal renal function [81], suggesting that the characterization and monitoring of these phenomena could be helpful from the earliest stages of the disease. The primarily fibrotic nature of the intermediate implies that this imaging parameter could be a promising marker for tracking disease progression in ADPKD patients. It might also help in predicting their long-term functional outcome.

Caroli et al. also found a strong correlation between the intermediate volume relative to the parenchyma, and both the GFR and GFR decline over a 3- to 8-year follow-up period in independent patient cohorts [72]. These findings, together with the previous ones [71], suggest that the most severe condition for a patient may be associated with the presence of large portions of fibrotic tissue relative to the preserved parenchyma, and highlight the intermediate volume potential for the early stratification of ADPKD patients, in addition to the monitoring of disease progression.

A few years later, Lai et al. used an advanced dynamic CE MRI protocol to identify the areas of the parenchyma with a normal perfusion and tissue architecture, called the “perfusion volume”, and the areas characterized by peritubular interstitial fibrosis, tubular dilation, atrophy and vascular sclerosis, called the “fibrotic volume”, in 15 ADPKD patients [73]. The presence of fibrotic volume was documented from the earliest stages of ADPKD. Moreover, both the total fibrotic volume, and the ratio between the total perfusion and kidney volume were significantly correlated with the eGFR, with a negative and positive association, respectively, suggesting these as possible new markers that could be used to monitor the disease progression and response to therapy.

A multicenter prospective study in ADPKD patients documented a closer relationship between the non-cystic kidney volume and renal function impairment than the TKV, on non-CE CT [74]. This study also found an association between radiomic features and renal function impairment, and showed that a combination of the non-cystic parenchyma and radiomic features had an excellent predictive value for renal function impairment.

More recently, Caroli et al. used diffusion-weighted MRI (DWI), a non-CE MRI modality enabling the investigation of the kidney microstructure [86], to identify and characterize the non-cystic kidney tissue component [75]. The non-cystic tissue showed a significantly higher diffusivity and lower pseudo-diffusion and flowing fraction than the healthy tissue. This is in line with the observation that ADPKD non-cystic tissue is not healthy, and includes fluid-filled microcysts that, despite not being visible on a macroscopic scale, do have a cyst-like microstructure [72], with a steady fluid where water molecules are free to diffuse (explaining the increased diffusivity). However, there is no observable flow, which explains the noted reduction in the pseudo-diffusion and flowing fraction parameters. This finding aligns with the documented presence of peritubular interstitial fibrosis in the non-cystic component, which has been observed from the earliest stages of the disease [72,73].

Along the same line, in the context of a multiparametric MRI study performed in 10 patients with early ADPKD, compared with 10 healthy young adults, Kline et al. reported significantly increased diffusivity values, and a decreased flowing fraction in the ADPKD non-cystic renal parenchyma [87]. Moreover, the authors also found significant differences between the ADPKD non-cystic renal parenchyma and normal renal tissue using magnetization transfer imaging (MTI), blood-oxygenation-level-dependent (BOLD) MRI, and magnetic resonance elastography (MRE) biomarkers, suggesting a multiparametric renal MRI potential for characterizing non-cystic kidney tissue in ADPKD.

Taken all together, these studies highlight the promise of the non-cystic parenchyma, particularly the fibrotic tissue component, as an early monitoring and prognostic biomarker in ADPKD. Special care should be taken regarding the imaging modality used to quantify and characterize non-cystic kidney tissue. Despite allowing the easy quantification of the non-cystic volume and separate perfused from the fibrotic tissue, CE-CT requires the administration of contrast media, and patient exposure to radiation, which come with not-negligible risks, especially for patients with an impaired renal function. Dynamic CE MRI does not entail exposure to radiation, but still requires the administration of a contrast medium. Non-CE MRI alternative modalities allowing the separation of the residual functioning parenchyma from the fibrotic/pathologic non-cystic component would be highly desirable.

## 5. Multiparametric Magnetic Resonance Imaging

In recent decades, several kidney MRI techniques have been proposed, to measure biophysical tissue properties linked to fibrosis, inflammation, tissue oedema, perfusion, filtration, and tissue oxygenation, beyond anatomy [88,89] (Table 1). In contrast to conventional MRI, which is limited to investigating gross anatomical changes, functional MRI allows the tissue to be characterized in detail. As such, quantitative MRI biomarkers may be able to pick up early signs of disease progression that have not yet led to a discernible effect on the markers in blood and urine. In addition, MRI biomarkers are unique among diagnostic tools, in that they characterize the entire kidney in high spatial detail, do not use ionizing radiation, and can assess the degree of functional heterogeneity across the kidney, thus showing the potential to improve the management of a range of kidney diseases, including ADPKD [89]. Despite the use of MRI in ADPKD being often limited to anatomical sequences enabling volume quantification, other MRI sequences have been used in preliminary studies to investigate ADPKD pathophysiology; namely, phase-contrast (PC) MRI, DWI, T2 mapping, and dynamic CE-MRI.

PC-MRI allows the non-invasive measurement of the renal blood flow (RBF) [90], and has been used in ADPKD since 2003. RBF measurement using PC-MRI has been shown to have a high accuracy and intra- and interobserver reproducibility in early ADPKD, to strongly correlate with both the renal volumes and GFR, and to predict GFR decline [96]. In a subsequent longitudinal ADPKD study, an RBF decrease over the 3-year follow-up was found to precede GFR decline, was negatively correlated with the TKV and total cyst volume slopes, and was positively correlated with the GFR slope, thus predicting the disease progression, and showing promise as outcome measure in ADPKD clinical trials [97]. PC-MRI was used in a small clinical trial to investigate the short-term effects of tolvaptan in patients with ADPKD, alongside the GFR and TKV; the study found no significant change in the RBF after one week of Tolvaptan treatment, using PC-MRI mirroring para-aminohippurate (PAH) clearance flow measurements [98]. More recently, Spithoven and colleagues provided additional evidence of the accuracy and validity of RBF measurement via PC-MRI, compared to RBF measured via continuous hippuran infusion. In this study, RBF values were associated with ADPKD severity, and technical problems preventing RBF measurement occurred predominantly in patients with a lower eGFR, suggesting that RBF measurement may be less feasible in patients with ADPKD at an advanced stage [99]. Lastly, PC-MRI-based RBF was used in combination with multiple other MR features, including hemorrhagic renal cysts, the renal cyst fraction, liver and spleen volume, and hepatic cyst fraction, to enhance the sensitivity for predicting eGFR decline in ADPKD, compared to the standard model including only the HtTKV [70].

Although DWI has been shown to be promising in several other kidney diseases [86], up to now, only a few studies have used DWI to investigate the kidney tissue microstructure in ADPKD. Suwabe and colleagues highlighted DWI’s sensitivity to intracystic infection, in spite of the low specificity [100]. Lupica and colleagues reported abnormally increased apparent diffusion coefficient (ADC) values and a reduced fractional anisotropy in ADPKD patients [101], despite no separation between the cystic and non-cystic components. More recently, as mentioned in the earlier sections, Caroli et al. used DWI to identify and characterize the non-cystic kidney tissue component [75], showing its higher diffusivity and lower pseudo-diffusion and flowing fraction than healthy tissue. Albeit preliminary, these studies highlighted DWI’s potential to characterise kidney tissue, and follow disease progression, in patients with ADPKD, from the earliest disease stages.

Renal magnetic resonance relaxometry, namely T1 and T2 mapping, has the potential to non-invasively investigate the kidney microstructure and function [91]. T2 mapping MRI has been used alongside DWI in an ADPKD preclinical study. The kidney T2 values and ADC were found to be highly sensitive markers of early cystogenesis in the ADPKD mouse model, to exhibit a nearly perfect correlation with the histological cystic index, and to be able to monitor the early treatment effects in a proof-of-principle experiment [102]. In the same study, a strong significant increase in T2 values was seen in early-stage ADPKD patients, compared with healthy volunteers. Based solely on T2 values, early-stage ADPKD patients with a kidney volume <300 mL could be distinguished from healthy volunteers, which was not possible based on the TKV. Along the same line, the T2 values in the residual parenchyma showed a strong association with disease severity in ADPKD patients from the earliest stages, and a higher correlation with the renal cyst fraction than the HtTKV [103].

Lastly, as mentioned earlier, dynamic CE MRI allowed the documentation of the presence of a fibrotic non-cystic volume from the earliest stages of ADPKD, and a significant correlation between the fibrotic volume and the eGFR [73], suggesting the potential of dynamic CE in monitoring disease progression and response to therapy in ADPKD.

**Figure 5:**
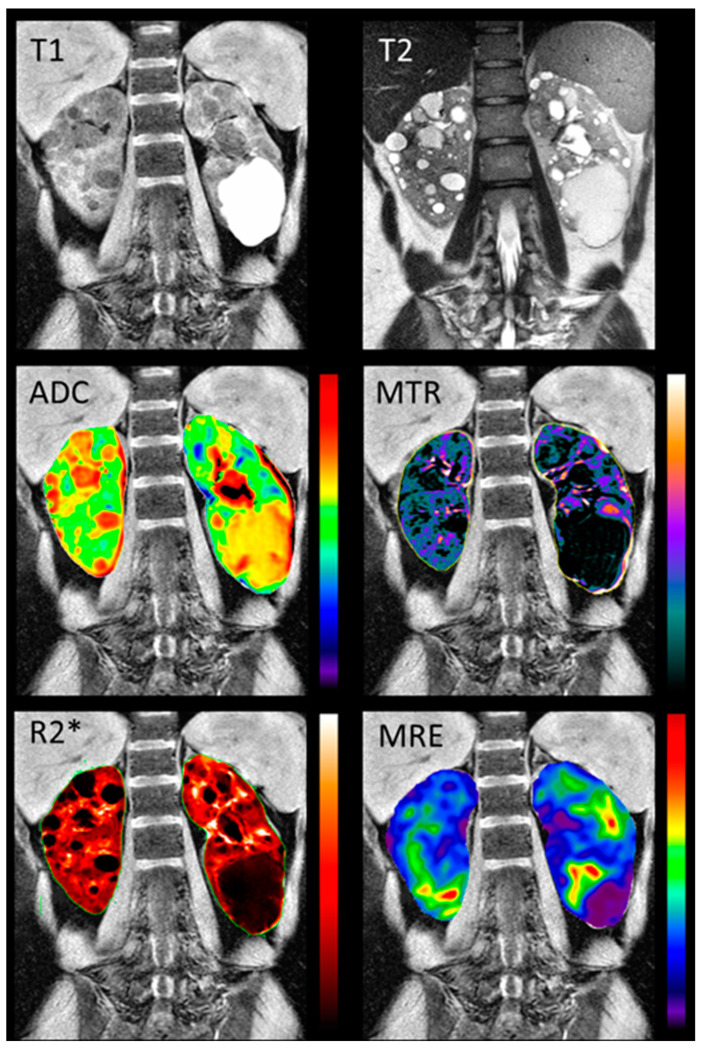
Examples of the multiparametric MRI of the kidney in a representative ADPKD patient. The top row shows structural MRI scans (T1- and T2-weighted). The second and third rows show quantitative maps, overlaid on the T1-weighted scan: the apparent diffusion coefficient ‘ADC’ derived from DWI, the magnetization transfer ratio ‘MTR’ derived from MTI, the apparent transverse relaxation rate ‘R2*’ derived from BOLD, and the tissue stiffness map derived from MRE. The figure was reprinted with permission from Kline, T.L. et al. Quantitative MRI of kidneys in renal disease [87].

To improve the specificity, and gain the most insight into kidney disease pathophysiology, individual MRI modalities are likely to benefit from their combination in a single acquisition session, through the so-called multiparametric MRI approach, therefore enabling a comprehensive characterization of the kidney tissue and function [104]. Two recent studies have highlighted the potential of averaging volumes obtained from different anatomical MRI sequences, following proper quality control, to mitigate possible TKV measurement bias, and thus improve the TKV reproducibility [105,106]. In addition, there is only one study so far that has used multiparametric renal MRI in ADPKD patients [87]. Comprehensive multi-parametric renal MRI scans, including T1-weighted, T2-weighted, FIESTA, 2D PC-MRI, DWI, MTI, BOLD MRI, and MRE (Figure 5) were acquired in 10 young adults with normal renal function, and 10 early ADPKD patients. The quantitative MRI sequences were found to be reproducible. Moreover, a significant difference between the ADPKD non cystic renal parenchyma and the normal renal tissue was found in the MTI, DWI, BOLD, and MRE biomarkers, suggesting the potential of multiparametric renal MRI in detecting and following renal disease from the earliest disease stages.

Despite multiparametric renal MRI showing promise in the non-invasive investigation of kidney pathophysiology in ADPKD, as well as in several other kidney diseases, it remains underused in clinical research. There is still a long way to go to achieve its translation to clinical practice, due to technical challenges, and the limited evidence of its biological and clinical validity. The studies published so far were performed on small samples of patients from single centers, and large multicenter longitudinal studies aimed at providing definitive evidence of biological and clinical validity are highly desirable. Moreover, the multiparametric approach involves a substantial number of MRI biomarkers, each requiring a non-negligible acquisition time, and poses the challenge of identifying the most informative biomarker combinations that are likely to be disease-area specific. Artificial intelligence algorithms and the most recent neural networks could be useful for this purpose. Lastly, the demonstration of cost-effectiveness, and the regulatory qualification of MRI biomarkers are necessary steps required for the successful translation of multiparametric MRI toward clinical practice [89].

## 6. Advanced Image Processing

**Figure 6:**
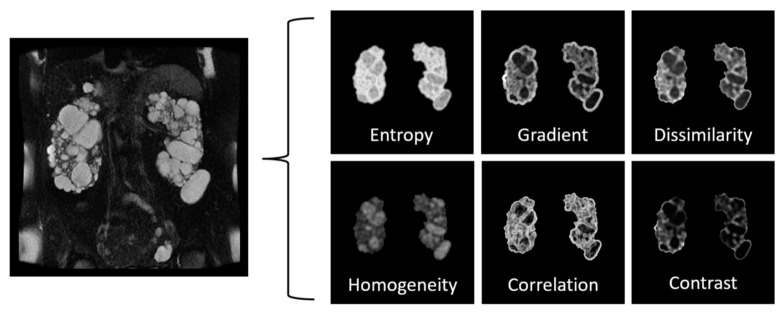
Radiomic feature extraction from a T2-weighted MRI scan of a patient affected by ADPKD. This figure elucidates the process of extracting radiomic texture features from a MRI T2-weighted scan. On the left panel, the ‘T2-Weighted Image’ represents the original scan. The right panel illustrates (as examples) the extraction of six key texture features, each displayed as an individual sub-panel: Entropy—a measure of the randomness or complexity in the image, which can reflect the heterogeneity within kidney tissues, Gradient—a descriptor of the rate of intensity change across the image, often highlighting the boundaries of cysts, Dissimilarity—providing an estimate of the variation or differences in intensity between neighboring pixels, Homogeneity—signifying the similarity in intensity values among adjacent pixels, and thereby indicating uniform regions, such as the interior of cysts, Correlation—which captures the linear relationship of gray levels in the image, assisting in identifying structured patterns within the tissue, and Contrast—providing a measure of the difference in intensity between a pixel and its neighbors, highlighting the edges and structural boundaries in the image. Each of these features offers valuable insights into the underlying tissue characteristics and disease processes in ADPKD, and underscores the potential of radiomics in advancing our understanding and management of this condition. Measurements of texture features within non-cystic regions may offer the potential to assess, for example, vasculature, fibrosis, and microscopic cysts. Measurements of texture features within the cyst regions may help in the identification of complex cysts (e.g., proteinaceous cysts) and/or malignancies.

Radiomics, also known as texture analysis, is a growing field that uses advanced image analysis techniques to extract quantitative information from medical images. Texture analysis involves the measurement of various image features, such as the intensity, shape, and spatial distribution of image structures. These features can provide valuable information about the heterogeneity of the cystic and non-cystic tissue, and can help to distinguish between simple and complex (e.g., proteinaceous) cysts. In the context of ADPKD, radiomics has been used to extract textural features from radiological images (Figure 6), and to correlate these features with the clinical severity of the disease.

In 2017, Kline et al. explored the potential of image texture features from MRI to enhance the predictive power of biomarkers for disease progression, in the context of ADPKD [107]. Using a retrospective cohort of 122 patients with T2-weighted MRIs and a normal eGFR, nine distinct image texture features were computed per patient. The features’ ability to predict progression to various stages of CKD, and a 30% reduction in the eGFR at the eight-year follow-up were assessed. A multiple linear regression model was developed, incorporating the age, baseline eGFR, HtTKV, and three image texture features (Entropy, Correlation, and Energy). The inclusion of texture in the model improved Pearson’s correlation coefficient from −0.51 to −0.70, suggesting that a texture analysis could refine ADPKD prognosis, and assist in individualized clinical decision-making and outcome prediction.

In 2021, Cong et al. aimed to develop and validate a radiomics method, based on the FS-T2WI MRI pulse sequence, for kidney function evaluation in patients with ADPKD [108]. Using clinical data and MRI images of 114 ADPKD patients, they extracted 960 radiomics features per volume of interest (VOI). Three models were constructed using pure clinical data, pure image data, and a combination of both. These were evaluated using five machine-learning classifiers. The clinical–image fused model outperformed the pure image model and the pure clinical model, demonstrating a superior diagnostic efficiency. Thus, the study concluded that MRI FS-T2WI radiomics analysis, based on a clinical–image fused model, is effective in evaluating and predicting kidney function in ADPKD patients.

A year later, Li et al.Ied a radiomics-based nomogram model to predict renal function in patients with ADPKD [109]. Using MRI data from 100 patients, they created a model using radiomics features, clinical factors, and conventional MRI variables. The nomogram model showed a superior predictive power compared to the basic radiomics model, demonstrating the potential for non-invasive renal function prediction, thus aiding in clinical decision-making for ADPKD patients.

Earlier this year, Kremer et al. conducted a study to identify texture-based differences in risk-stratified Mayo Imaging Classification (MIC) groups in ADPKD, and to find the optimal pre-processing parameters for feature extraction [110]. The study used T2-weighted fat-saturated MRI scans from 128 patients, categorized into low/intermediate- and high-risk MIC classes. Features were extracted from the non-cystic kidney parenchyma and the entire kidney, using different levels of gray-level discretization and pixel resampling. The least absolute shrinkage operator (LASSO) combined the relevant features into a logistic regression model. The study found that in the area under the receiver operating characteristic curve (AUC), the values ranged from 0.68–0.84 for the non-cystic kidney, and 0.83–0.88 for the entire kidney. The results suggest that texture-based differences among the risk-stratified MIC classes in both the non-cystic and entire kidney parenchyma can help better identify patients at risk of end-stage kidney disease.

While the outcomes of radiomics studies in ADPKD indeed show potential, it is crucial to acknowledge that these are initial findings, and that comprehensive research is still required to definitively determine the clinical value of these methods. Furthermore, it is important to affirm these techniques through validation studies, before they can be routinely incorporated into everyday clinical practice.

The successful widespread implementation of radiomics in ADPKD management hinges on several factors. A key aspect is the development of standardized protocols for radiomics analysis. Having a clear, consistent, and universally accepted approach to the analysis would ensure that the results were comparable across different studies and settings, thereby increasing the reliability and acceptance of the results.

Another vital component is the availability of validated software tools. Given the complexity of radiomics analysis, it is important to have software tools that are not only efficient and accurate, but also have been rigorously tested and validated. This would ensure that the analysis was performed correctly, and that the results could be trusted. As we move forward, we must focus on these critical aspects, to ensure the successful implementation of this promising new technique.

In summary, while the initial results indicate a potential game-changer in the management of ADPKD, the journey toward integrating radiomics into standard practice requires robust research and validation efforts. Nevertheless, the promise of these innovative techniques is undeniably exciting, and opens up new possibilities for improving ADPKD management.

## 7. Conclusions and Future Directions

ADPKD is a prevalent hereditary renal disease, requiring early biomarkers to detect and predict disease progression. Renal enlargement, a key aspect of ADPKD, has been assessed using imaging techniques such as CT, MRI, and US, each denoted by different pros and cons (Table 2).

This review presented an in-depth analysis of the current state of the art of diagnostic imaging in ADPKD, emphasizing the importance of various imaging biomarkers, from established methods such as total kidney and cyst volume measurements, to innovative approaches for tracking disease progression. Furthermore, this review discusses the latest advancements in artificial intelligence for quantifying imaging biomarkers, and the potential of multiparametric approaches to provide enhanced monitoring of ADPKD. These biomarkers are vital for clinical monitoring, and for evaluating new treatment strategies in clinical trials, with the ultimate aim of improving the prognosis and management of ADPKD patients. Advanced image processing techniques, such as radiomics and texture analysis, hold immense potential to revolutionize the characterization and management of ADPKD. Radiomics/texture analysis can extract a high-dimensional set of quantitative features from medical images, which may provide a more detailed insight into the disease’s heterogeneity. These approaches could also identify subtle patterns not discernible by the human eye, potentially serving as early indicators of disease progression.

The use of multi-modal data is another promising avenue to be explored. By integrating data from various imaging modalities (such as CT, MRI, and US) with clinical, genetic, and proteomic data, we could develop a more comprehensive characterization of ADPKD. This integrative approach could enhance the accuracy of disease prognosis, and the personalization of treatment strategies.

Advanced image acquisitions, such as diffusion-weighted imaging, magnetization transfer, arterial spin labeling, and BOLD MRI, with no risk to the patient, could provide further functional and structural insights into the ADPKD kidney. These techniques can offer additional information about renal blood flow, oxygenation, and microstructural changes, which may allow the characterization of the non-cystic kidney tissue microstructure in addition to cyst expansion, and thus be particularly valuable from the early stages of the disease.

Furthermore, advanced AI-based approaches, such as deep learning, could be utilized in automated and more accurate image interpretation, early diagnosis, and prediction of disease progression. One could envision the use of AI, not just in cyst segmentation, but in the prediction of cyst formation and growth, based on early imaging and clinical data.

Additionally, the utilization of machine-learning models could facilitate the discovery of new imaging biomarkers, and the identification of complex patterns within multi-modal data. AI could also be harnessed to predict patient outcomes and responses to treatments, enhancing the precision of clinical decision-making in ADPKD management.

The exploration of additional novel imaging techniques, such as contrast-enhanced ultrasound (CEUS), dual-energy CT (DECT), 3D phase-contrast MRI (i.e., 4D Flow), and molecular imaging techniques, such as positron emission tomography (PET), could help further enhance our understanding and management of ADPKD. CEUS could be a safer alternative to contrast-enhanced CT and/or MRI, especially for patients with renal impairment. In addition, CEUS could potentially help to assess the renal blood flow, and detect complications related to cysts, such as infections or neoplasms. On the other hand, DECT could provide more detailed information about tissue composition, compared to standard CT. In ADPKD, it might help to identify calcifications, and/or perform stone composition analysis. The 4D flow MRI technique could be used to assess changes in the renal blood flow (and potentially automate/standardize the measurements), and reduce the interoperator variability (e.g., as seen in 2D phase contrast methods). Lastly, PET could be used to explore metabolic changes in kidney tissues, potentially providing insights into disease mechanisms and progression.

Although a high level of focus has been given to MRI, US and CT continue to provide important information in the study and care of patients affected by ADPKD. Ultrasound, while less precise in computing the TKV compared to advanced imaging techniques [111,112], remains of value in the diagnosis of ADPKD, especially in screening the at-risk children of parents affected by ADPKD [113]. Recent studies [56,114,115] emphasize the relevance of ultrasound in pediatric populations, and its potential in assessing the renal resistive index in ADPKD.

Similarly, while contrast-enhanced CT offers a higher resolution than ultrasound, is suitable for computing the TKV and cyst volumes, and is the best modality for detecting renal calcifications, including stones causing ureteral obstruction, which are more common in ADPKD compared to the general population; it poses potential risks, due to radiation exposure. Moreover, the need for iodinated contrast media is a significant concern for patients with reduced renal function. The inherent limitations of US and CT, along with the versatile capabilities of MRI, have indeed driven the broader scientific community to focus more on MRI in the context of ADPKD.

In addition to the previously discussed imaging biomarkers, recent research has illuminated the potential of urinary and metabolic biomarkers in ADPKD. For instance, emerging urinary markers, such as the urinary excretion of β2MG, and MCP-1 excretion [116], are associated with GFR decline in ADPKD. Metabolic markers, including changes in urinary metabolomic profiles [117], provide further biochemical insights into the disease’s progression and therapeutic response. The strength of advanced imaging biomarkers lies in their ability to capture early morphological changes in the kidneys, possibly before noticeable impacts on the blood and urine markers. Nevertheless, the value of a multimodal approach, integrating both advanced imaging and conventional urinary biomarkers, is becoming increasingly recognized. Such an approach can deliver a more comprehensive understanding of disease progression, catering to the heterogeneous nature of ADPKD, and potentially informing individualized management strategies.

The creation of ADPKD nomograms is another innovative concept worth exploring. These graphical calculation tools would use multi-modal data to predict individual patient outcomes, providing a personalized prognosis based on various factors, such as the cyst size, growth rate, genetic mutations, and protein levels. These nomograms would require advanced machine-learning algorithms for development and, once validated, could provide a major leap forward in personalized ADPKD treatment. Many existing calculators exist and, through techniques such as federated learning, models could be developed utilizing multi-institutional data, while maintaining data security and privacy.

Lastly, any imaging biomarker is useful in ADPKD patient management only if it can enter clinical practice, showing feasibility and effectiveness in the clinical setting. The available imaging modalities show different advantages and disadvantages, according to their purpose of use (Table 3).

Improved imaging methods are needed to ensure: (i) a better prognostic accuracy, considering ADPKD’s variable progression rate between individuals; (ii) the earlier detection of disease progression, allowing for timely interventions; (iii) the monitoring of the treatment response, and (iv) improved cost-effectiveness and safety.

From the initial phase, novel imaging biomarkers should be developed with an eye toward regulatory approval, and compliance with clinical practice. In this respect, AI-based algorithms could be transformative, although would come with clinical implementation challenges, including generalizability, and the need for the education of interdisciplinary stakeholders. After approval by regulatory agencies, and successful examples of the clinical implementation of an AI-based computation algorithm, the TKV is undoubtedly the imaging biomarker closest to clinical routine, and should serve as a model for all other ADPKD imaging biomarkers. In conclusion, the future of ADPKD characterization and management lies in harnessing the power of advanced imaging, multi-modal data integration, and AI techniques. These strategies will potentially enable earlier diagnosis, personalized treatment, and improved patient outcomes. In general, further research is needed to validate these techniques, and to work toward translating them into clinical practice.

## Figures and Tables

**Table 1:** The most common quantitative MRI measures that may be combined within a multiparametric renal MRI protocol, and could also be informative for ADPKD.

MRI Measure	Description
Diffusion weighted imaging (DWI)	Provides information about the kidney microstructure by detecting the displacement of water molecules (Brownian motion) within the architecture of tissues [86].
Phase-contrast (PC-MRI)	Measures the blood flow in the renal arteries [90].
T1 mapping	Provides a quantitative map over the whole kidney for the T1 values, giving information on the kidney microstructure [91].
T2 mapping	Provides the quantification of T2 as a tissue-specific time parameter [91].
Blood-oxygen-level-dependent (BOLD) MRI	Indirectly assesses renal oxygenation, exploiting the paramagnetic properties of deoxygenated hemoglobin [92].
Arterial spin labelling (ASL)	Measures tissue perfusion using magnetically labelled water protons in the blood [93].
Magnetization transfer imaging (MTI)	Provides information about the tissue composition and microstructure by measuring the magnetization transfer ratio.
Magnetic resonance elastography (MRE)	Provides information about the kidney tissue stiffness, using shear waves propagating in the tissues [94].
Dynamic contrast-enhanced MRI	Measures renal perfusion and provides a direct measure of the GFR, although it requires the administration of an exogenous contrast agent [95].

**Table 2:** The value and limitations of imaging methods/techniques available to scan ADPKD patients.

	US	Non-Contrast Enhanced CT	Contrast Enhanced CT	Traditional MRI	Advanced/ mp MRI
**Availability**	Very high	High	High	Average	Low
**Radiation exposure**	No	No	Yes	No	No
**Contrast agent**	No	No	Iodinated contrast media. Rare allergic reaction. Few side-effects. Risk of contrast-induced nephropathy (GFR < 60 mL/min)	No	Gd-based contrast media (for DCE and molecular MRI only). Very rare allergic reactions. Risk of NSF, older-generation agents contraindicated in patients with GFR < 30 mL/min. Gd retention in the brain
**Acquisition time**	Short	Very short	Short	Average	Very long
**Spatial resolution**	Very low	High	Very high	High	High
**Versatility**	Low	Low	Low	Low	Very high
**Clinical contraindications**	None	Pregnancy, claustrophobia	Pregnancy, hyperthyroidism, claustrophobia	Metal implants or devices, presence of foreign bodies, claustrophobia	Metal implants or devices, presence of foreign bodies, claustrophobia
**Cost**	Very low	Average	Average	High	High
**Patient weight limit**	No	Yes	Yes	Yes	Yes
**Operator-dependence**	Very high	Low	Low	Quite low	Quite low
**Patient comfort**	Very high	Quite high	Quite high	Quite low	Low

Abbreviations: US = ultrasound, CT = computed tomography, MRI = magnetic resonance imaging, GFR = glomerular filtration rate, Gd = gadolinium, DCE = dynamic contrast-enhanced, NSF = nephrogenic systemic fibrosis, mp = multiparametric.

**Table 3:** Advantages and disadvantages of each imaging modality, from diagnosis to prognosis.

	Diagnosis	Staging	Monitoring (Disease Progression/ Response to Therapy)	Prognosis
**US**	Identification and counting of cysts and rough estimation of TKV	Rough estimation of TKV	Not accurate (low resolution)	Not accurate (low resolution)
**Non-contrast-enhanced CT**	Reliable quantification of TKV but not of cyst number	Reliable quantification of TKV	Reliable assessment of TKV change over time	Reliable quantification of TKV (validated prognostic marker)
**Contrast-** **enhanced CT**	Reliable quantification of TKV and accurate cyst counting	Reliable quantification of TKV, TCV, and the volume of non-cystic pathologic/fibrotic component	Reliable assessment of TKV, TCV, and non-cystic pathologic/fibrotic volume change over time	Reliable quantification of TKV (validated prognostic marker), as well as TCV and non-cystic pathologic/fibrotic volume (showing potential as prognostic markers)
**Traditional MRI**	Reliable quantification of TKV and accurate cyst counting	Reliable quantification of TKV and TCV	Reliable assessment of TKV and TCV change over time	Reliable quantification of TKV (validated prognostic marker), as well as TCV
**Advanced/** **mp MRI**	Reliable quantification of TKV and accurate cyst counting.	Reliable quantification of TKV and TCV, and identification and characterization of the non-cystic pathologic/fibrotic component (both microstructure and function)	Reliable assessment of TKV, TCV, and non-cystic pathologic/fibrotic volume change over time	Reliable quantification of TKV (validated prognostic marker) and TCV, as well as characterization of the non-cystic pathologic/fibrotic volume (showing potential as prognostic marker)

Abbreviations: Abbreviations: US = ultrasound, CT = computed tomography, MRI = magnetic resonance imaging, TKV = total kidney volume; TCV = total cyst volume, mp = multiparametric.
